# Serum D-lactate, a novel serological biomarker, is promising for the diagnosis of periprosthetic joint infection

**DOI:** 10.1186/s12891-022-05199-8

**Published:** 2022-03-26

**Authors:** Yanyang Chen, Huhu Wang, Xiyao Chen, Hairong Ma, Jingjie Zheng, Li Cao

**Affiliations:** Department of Orthopaedics, First Affiliated Hospital of Xinjiang Medical University, No.137 South Li Yu Shan Road, Urumqi, 830054 Xinjiang China

**Keywords:** Joint arthroplasty, Periprosthetic joint infection, Diagnosis, Serum D-lactate

## Abstract

**Background:**

Although many markers are used for diagnosis of periprosthetic joint infection (PJI), serological screening and diagnosis for PJI are still challenging. We evaluated the performance of serum D-lactate and compared it with ESR, coagulation-related biomarkers and synovial D-lactate for the diagnosis of PJI.

**Methods:**

Consecutive patients with preoperative blood and intraoperative joint aspiration of a prosthetic hip or knee joint before revision arthroplasty were prospectively included. The diagnosis of PJI was based on the criteria of the Musculoskeletal Infection Society, and the diagnostic values of markers were estimated based on receiver operating characteristic (ROC) curves by maximizing sensitivity and specificity using optimal cutoff values.

**Results:**

Of 52 patients, 26 (50%) were diagnosed with PJI, and 26 (50%) were diagnosed with aseptic failure. ROC curves showed that serum D-lactate, fibrinogen (FIB) and ESR had equal areas under the curve (AUCs) of 0.80, followed by D-dimer and fibrin degradation product, which had AUCs of 0.67 and 0.69, respectively. Serum D-lactate had the highest sensitivity of 88.46% at the optimal threshold of 1.14 mmol/L, followed by FIB and ESR, with sensitivities of 80.77% and 73.08%, respectively, while there were no significant differences in specificity (73.08%, 73.08% and 76.92%, respectively).

**Conclusion:**

Serum D-lactate showed similar performance to FIB and ESR for diagnosis of PJI. The advantages of serum D-lactate are pathogen-specific, highly sensitive, minimally invasive and rapidly available making serum D-lactate useful as a point-of-care screening test for PJI.

## Introduction

Periprosthetic joint infection (PJI) is a devastating complication with severe health and socioeconomic implications that occurs after total joint arthroplasty, with an incidence rate of approximately 1% to 3% in patients undergoing primary total joint arthroplasty and 3% to 5% in those undergoing revision total joint arthroplasty [1, 2]. Furthermore, missed diagnosis or the misdiagnosis of PJI can result in endless hospital stays and overtaxed medical care and can also cause higher mortality and morbidity [3]. Therefore, a timely preoperative and accurate diagnosis of PJI is extremely important.

Unfortunately, there is still no absolute test for the diagnosis of PJI, compelling the Musculoskeletal Infection Society (MSIS) to formulate stepwise diagnostic criteria [4, 5]. However, the MSIS criteria would probably lead to diagnosis or misdiagnosis in the first step of evaluating serum C-reactive protein (CRP) and erythrocyte sedimentation rate (ESR) which have limited value for screening and diagnosing PJI due to their unsatisfying sensitivity and reliability, when facing a considerable number of patients infected by slow-growing organisms, in early postoperative periods, or with a low level of inflammation and subtle clinical symptoms [6–9]. Thus, novel diagnostic tests with better sensitivity and reliability are required. However, more attempts have been made to investigate synovial fluid instead of blood, whereas serological tests would be simpler and more practical and could act as the first hurdle.

Most recently, several studies have demonstrated that D-lactate is a promising biomarker for diagnosing patients with sepsis and meningitis, including in patients receiving antimicrobial therapy, although the measure of the D-lactate concentration in primary sterile body fluids for discriminating infection from aseptic inflammation could back in the 1990s [10–16]. D-lactate, an isomer of L-lactate, with a molecular weight of 90 Da, is the predominant form of lactate produced by different bacterial species and fungi [17, 18]. And D-lactate was the product of pyruvate which was catalyzed using D-lactate dehydrogenase with NADH as coenzyme [19]. In mammals,

D-lactate appears to be present in very low levels in serum (it is mainly produced by intestinal bacteria and their absorption). As human cells can only produce L-lactate but not D-lactate. D-lactate cannot be metabolized in mammalian tissues due to the lack of D-lactate dehydrogenase and the low renal clearance rate. However, bacterias have the ability to produce both D-lactate and L-lactate, an elevated serum D-lactate may suggest a bacterial infection [18, 20].

As coagulation-related indicators are linked closely to infection, several coagulation biomarkers, such as fibrinogen degradation products (FDPs), D-dimer and fibrinogen (FIB), are reported as good biomarkers for PJI diagnosis [12, 21]. Thus, the aim of this study was to evaluate the performance of serum D-lactate as an independent diagnostic marker for PJI and compare it with conventional inflammatory ESR, coagulation-related biomarkers in serum and synovial D-lactate.

## Materials and Methods

### Study design and patients

This prospective case-control study started from June 2020 to February 2021, and 26 total knee arthroplasty (TKA) or total hip arthroplasty (THA) PJI patients diagnosed according to the MSIS criteria shown in Table 1 were enrolled as the PJI group. Twenty-six aseptic TKA or THA failure patients who were matched to PJI patients by age and sex were enrolled as the control group. The baseline data, including sex, age, body mass index (BMI), and involved joints, are shown in Table 2. The exclusion criteria were as follows: patients with insufficient fluid volume for analysis of D-lactate (<1 mL), bacterial infection outside the joint a history of recent trauma, hematoma, skin ulcers, ecchymosis, heart valve prosthesis, or dysfunction of coagulation.

**Table 1:** MSIS Criteria for the Diagnosis of PJI and Thresholds for the Minor Diagnostic Criteria^a^

		Recommended Threshold
Major criteria	(1) Two positive periprosthetic cultures with phenotypically identical microorganisms or (2) A sinus tract communicating with the joint	
Minor criteria	(1) Elevated serum CRP and ESR	10 mg/L, 30 mm/h
	(2) Elevated SF WBC count or changes in the leukocyte esterase strip	3,000 cells/μl, + or ++
	(3) Elevated SF PMN%	80%
	(4) Positive histological analysis of the periprosthetic tissue	>5 neutrophils per high-power
	(5) A single positive culture	field in 5 high-power fields (×400)

^a^PJI is present when 1 of the major criteria, or 3 of 5 of the minor criteria, are met. *MSIS* Musculoskeletal Infection Society, *CRP* C-reactive protein, *ESR* Erythrocyte sedimentation rate, *SF* Synovial fluid, *WBC* White blood cell, *PMN%* Polymorphonuclear neutrophil percentage

**Table 2:** Baseline characteristic of PJI and Aseptic patients

	PJI (*n*=26)	Aseptic (26)	Statistics tests	*P* -value
Gender, n (%)			*χ*^*2*^=0.308	0.579
Male	14(53.85)	12(46.15)		
Female	12(46.15)	14(53.85)		
Age(y), mean ±SD	61.62±10.71	63.96±11.41	*t*= - 0.76	0.45
BMI (kg/m^2^), mean ±SD	25.81±5.00	25.05±3.61	*t*=0.60	0.56
Joint, n (%)			*χ*^*2*^=6.24	0.01*
Hip	9(34.62)	18(69.23)		
Knee	17(65.34)	8(30.77)		

*PJI* Periprosthetic joint infection, *BMI* Body mass indexm, *SD* Standard deviation

* P<0.05

### Specimen collection and testing

Blood samples for detecting ESR and coagulation-related indicators were collected as part of the routine examination after admission. Then all were sent to the medical laboratory for detection as soon as possible. The ESR was detected using Westergren methods, and coagulation was quantified with a Sysmex CA-500 automatic blood coagulation analyzer (Sysmex, Japan). Synovial fluid was extracted before opening the joint capsule during revision surgery and then using a Sysmex XN2000 hematology analyzer (Sysmex, Japan) to determine synovial fluid (SF) white blood cell (WBC) count and the polymorphonuclear neutrophil percentage (PMN%). For D-lactate, one milliliter of blood and synovial fluid were collected from each sample above. After centrifugation, the supernatant was collected and stored in a refrigerator at -80°C until the final tests could be conducted uniformly.

### Bacterial culture and identification

After incubating for 12-18 hours at 37°C for enrichment culture, the specimens was inoculated with the inoculated ring and inoculated in the blood plate with streaking. After culture at 35°C for 24-48 hours, a single colony was selected for Gram staining. Then, Gram-positive bacteria were inoculated on blood agar and gram-negative bacteria were inoculated on blood agar or McConkey Agar, and individual colonies were identified using Matrix-assisted laser desorption ionization-time of flight mass spectrometer (Biomrieux, France). The results are shown in Table 3.

**Table 3:** Culture results of PJI patients(*n*=26) without possible bias

Culture Results	Number of patients
Positive	17
Staphylococcus epidemidis	7
Staphylococcus aureus	4
Enterobacter cloacae	1
Streptococcus sanguis	1
Corynebacterium stearate tuberculosis	1
Streptococcus hemolyticus	1
Granulicatella adiacens	1
Beehive bacillus	1
Negative	9

*PJI* Periprosthetic joint infection

### D-lactate ELISA

Serum and synovial D-lactate levels were measured with an ELISA Kit from Shuang Ying Biological Technology Co., Ltd., Shanghai, China. This D-lactate ELISA kit utilized a double-antibody one-step sandwich method, and the concentration was determined by a spectrophotometric method using a standard microplate absorbance reader at 450 nm. Six graded concentrations and the related optical density (OD) of the standard substance were utilized to draw a linear regression standard curve (R^2^ > 0.99). Each sample concentration was determined based on comparing their own OD to the standard curve. All experimental procedures were carried out according to the manufacturer’s instructions. The minimum detection concentration was less than 0.05 mmol/L. The intra- and inter-assay coefficients of variation (CVs) were less than 15%.

### Statistical analysis

Frequencies and chi-square tests were utilized for qualitative data. Means with standard deviations (SD) were described for normally distributed data, and medians with quartiles were calculated for abnormally distributed data. Student’s t-test and the Mann-Whitney test were performed to compare different biomarkers between the two groups. ROC curves and AUCs were utilized to compare the diagnostic performance of different biomarkers. The AUC values were considered excellent (0.900-1.000), good (0.800-0.899), fair (0.700-0.799), poor (0.600-0.699) or to have no discriminatory capacity (0.500-0.599). Sensitivity, specificity, positive predictive value (PPV) and negative predictive value (NPV) were calculated at the optimal cutoff value based on the Youden index. All statistical analyses were performed using SPSS version 24 (IBM Inc., USA) and GraphPad Prism 7.0a (GraphPad Software, USA). A *P*-value of 0.05 was considered statistically significant.

## Results

### Patient demographic data

The clinical characteristics of the PJI and aseptic groups are presented in Table 2. We included a total of 26 PJI cases and 26 cases without infection, including 27 (52%) hip and 25 (48%) knee prostheses in our study. There were no significant differences in sex, age or BMI between the PJI group and the aseptic group (*p* > 0.05). All patients have undergone revision surgeries.

### Comparison of indicators between the PJI group and the aseptic group

We compared the values of coagulation-related indicators, ESR, serum D-lactate, synovial D-lactate, SF WBC count and SF PMN%, and the results are shown in Table 4. There were slight increases in prothrombin time (PT), international normalized ratio (INR) and activated partial thromboplastin time (APTT) and significant increases in FIB, ESR, serum D-lactate, synovial D-lactate, SF WBC count and SF PMN% (*p* < 0.05), while there was no significant difference in the coagulation biomarker of thrombin time (TT) between the PJI group and the aseptic group (*p* > 0.05).

**Table 4:** Comparison of different biomarkers between PJI and aseptic failures

Biomarkers	PJI(*n*=26)	Aseptic(*n*=26)	Statistic tests	*P*
TT(s)^b^	19.42 ± 2.04	19.85 ± 1.30	*t=* - 0.892	0.377
PT(s)^a^	11.90(11.38 - 12.93)	11.40(11.25 - 11.83)	Z= - 2.310	0.021*
INR^a^	1.04(0.98 - 1.11)	0.99(0.97 - 1.03)	Z= - 2.283	0.022*
APTT (s)^a^	32.95(31.30 - 35.83)	30.95(29.68 - 32.70)	Z= - 2.608	0.009*
FDP (ug/ml)^a^	3.66(2.38 - 6.55)	1.92(1.14 - 3.75)	*Z= -* 2.270	0.023*
D-dimer (ng/ml)^a^	530.00(257.50 - 886.50)	248.00(110.50 - 610.50)	*Z= -* 2.115	0.034*
FIB (g/L)^a^	4.10(3.48 - 4.48)	3.01(2.54 - 3.57)	*Z*= - 3.615	0.000*
ESR (mm/h)	50.00(30.00 – 68.00)	20.00(14.00 – 32.00)	*Z= -* 3.718	0.000*
Serum D-lactate (mmol/L)^a^	1.70(1.45 - 2.33)	0.44(0.60 - 1.33)	*Z= -* 3.651	0.000*
Synovial D-lactate (mmol/L)^b^	2.43 ± 0.58	1.29 ± 0.83	*t=* 5.530	0.000*
SF WBC count (cells/μl)^a^	12174.00(5622.00 – 26347.00)	2365.00(1339.00 – 9706.50)	*Z= -* 3.318	0.001*
SF PMN%^a^	95.00(90.00 – 96.50)	51.65(40.00 – 79.25)	*Z= -* 4.315	0.000*

*PJI* Periprosthetic joint infection, *TT* Thrombin time, *PT* Prothrombin time, *INR* International normalized ratio, *APTT* Activated partial thromboplastin time, *FDP* Fibrinogen degradation products, *FIB* Fibrinogen, *ESR* Erythrocyte sedimentation rate, *SF* synovial fluid, *WBC* White blood cell, *PMN%* Polymorphonuclear neutrophil percentage

^a^The data was shown as median (interquartile range)

^b^The data was expressed as mean ±SD. SD, standard deviation

* *P*<0.05

### Performance of serum D-lactate

**Fig. 1:**
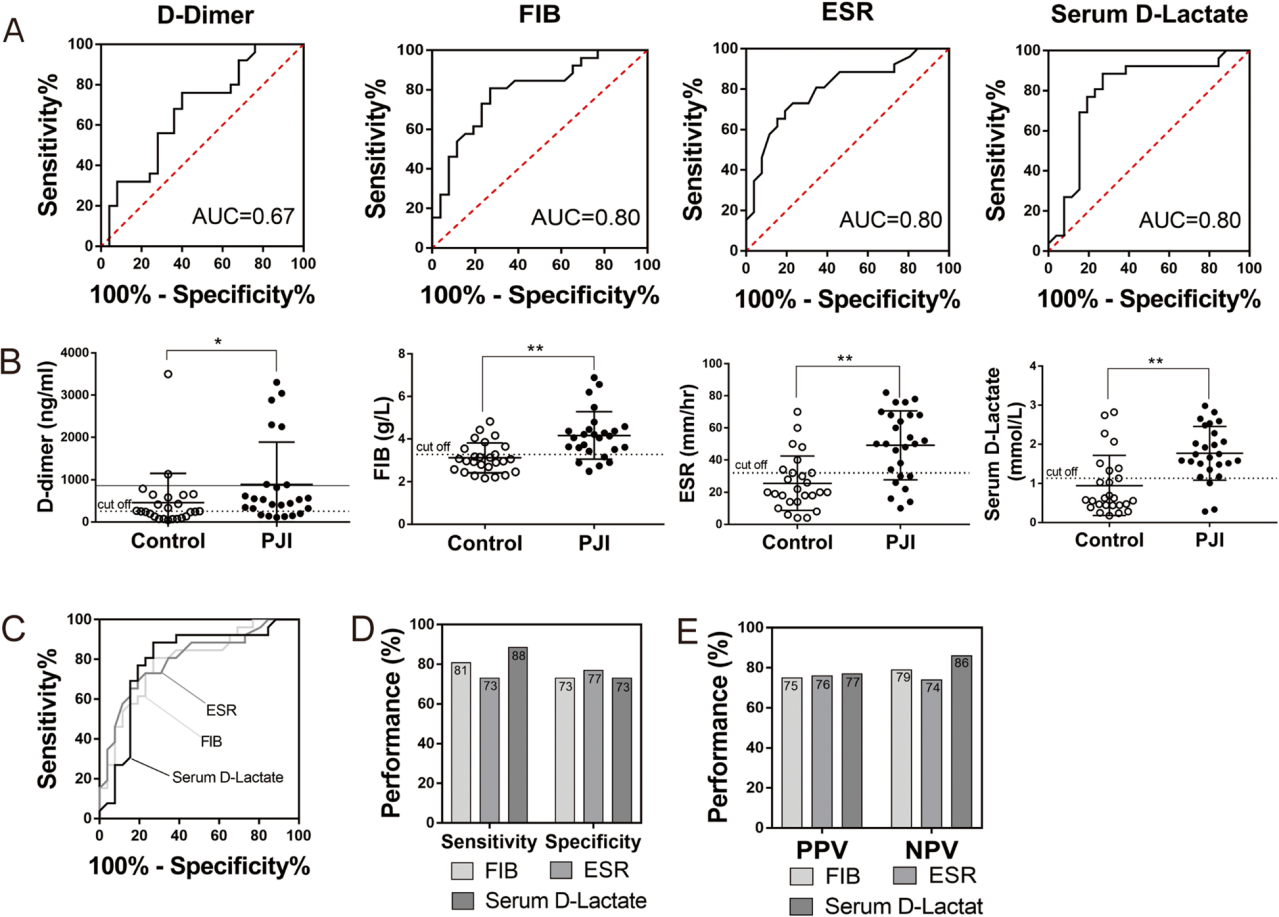
Comparison of serum D-lactate with FIB, ESR and D-dimer. **A** and **C** The ROC curves of serum biomarkers for the diagnosis of PJI. The AUC of D-dimer, serum D-lactate, FIB and ESR 0.67, 0.80, 0.80 and 0.80, respectively. **B** The distributions (median and IQR) of D-dimer, D-lactate, FIB and ESR in PJI and aseptic group. Solid lines represent the cutoff value recommended in a previous study8. Dotted lines represent the optimal. Threshold based on the current study. **D** Sensitivity and specificity of D-lactate, FIB and ESR. (E) PPV and NPV of D-lactate, FIB and ESR

The AUC of serum D-lactate was 0.80 when 1.14 mmol/L was chosen as the optimal cutoff value. The sensitivity and specificity of the D-lactate test were 88.46% and 73.08%, respectively. In 7 cases of aseptic failure, D-lactate concentrations increased above the cutoff, including 5 aseptic cases with ESR under the threshold, and the other 2 cases were also false positive in synovial D-lactate and ESR. Three patients were diagnosed with PJI according to the applied definition criteria and showed negative results for D-lactate. Of these, one case was diagnosed with a sinus tract, the second was confirmed by finding *Streptococcus sanguis* in the prosthesis ultrasonic vibration fluid, and the last was based on multiple fulfilled criteria (*p* < 0.001) (Table 5) (Fig 1A and B).

**Table 5:** Performance of serological tests for diagnosing PJI

	AUC (95% CI)	Youden index (95% CI)	Optimal Cut-off (95%CI)	Sensitivity (%)	Specificity (%)	PPV (%)	NPV (%)
PT (s)	0.69 (0.54 - 0.81)	0.38 (0.19 - 0.58)	12.10 (11.70 - 12.80)	46.15	92.31	85.71	63.16
INR	0.68 (0.54 - 0.81)	0.35 (0.15 - 0.50)	1.06 (0.99 - 1.07)	38.46	96.15	90.91	60.98
APTT (s)	0.71 (0.57 - 0.83)	0.42 (0.19 - 0.62)	32.20 (31.00 - 36.20)	69.23	73.08	72.00	70.37
FDP (ug/ml)	0.69 (0.54 - 0.81)	0.40 (0.160 - 0.60)	2.46 (1.92 - 7.79)	76.00	64.00	67.86	70.83
D-dimer (ng/ml)	0.67 (0.53 - 0.80)	0.36 (0.16 - 0.56)	265.00 (94.00 - 2342.14)	76.00	60.00	65.52	69.57
FIB (g/L)	0.80 (0.66 - 0.90)	0.54 (0.30 - 0.70)	3.27 (3.12 - 4.31)	80.77	73.08	75.00	79.17
ESR (mm/h)	0.80 (0.67 - 0.90)	0.50 (0.19 - 0.65)	32.00 (20.00 – 48.00)	73.08	76.92	76.00	74.07
Serum D-lactate (mmol/L)	0.80 (0.66 - 0.90)	0.62 (0.38 - 0.77)	1.14 (0.69 - 1.51)	88.46	73.08	76.70	86.40

*PJI* Periprosthetic joint infection, *AUC* Area under the curve, *CI* Confidence interval, *PT* Prothrombin time, *INR* International normalized ratio, *APTT* Activated partial thromboplastin time, *FDP* Fibrinogen degradation products, *FIB* Fibrinogen, *ESR* Erythrocyte sedimentation rate, *PPV* Positive predictive value, *NPV* Negative predictive value

### Comparison of serum D-lactate with ESR and coagulation biomarkers

Based on our data, the ROC curves showed that FIB, ESR and serum D-lactate had the highest AUC, at 0.80, indicating that they had good performance in the diagnosis of PJI (Table 5) (Fig 1 A and B). Although the AUC values for FIB, ESR and serum D-lactate were the same, serum D-lactate had the highest sensitivity and NPV of 88.46% and 86.40% at the optimal threshold of 1.14 mmol/L, followed by FIB and ESR, which had a sensitivity and NPV of 80.77% and 79.17% at the optimal threshold of 3.27 g/L and 73.08% and 74.07% at the optimal threshold of 32.00 mmol/h, respectively. In addition, no significant differences in specificity or PPV were observed in FIB, ESR and serum D-lactate, the distribution of which is depicted in Table 5 and Fig 1C, D and E.

The other coagulation biomarkers of PT, INR, APTT, FDP and D-dimer did not have ideal AUCs, at 0.69, 0.68, 0.71, 0.69 and 0.67, respectively, indicating that they are of limited value for diagnosing PJI. In particular, for the novel biomarker D-dimer, the optimal threshold was 265.00 ng/ml, with a sensitivity, specificity, PPV, and NPV of 76.00%, 60.00%, 65.52% and 69.57%, respectively. For FDP, the optimal threshold was 2.46 μg/ml, which resulted in a sensitivity, specificity, PPV, and NPV of 76.00%, 64.00%, 67.86% and 70.83%, respectively. (Table 5).

### Comparison of synovial D-lactate with leukocyte count and polymorphonuclear neutrophil percentage

**Fig. 2:**
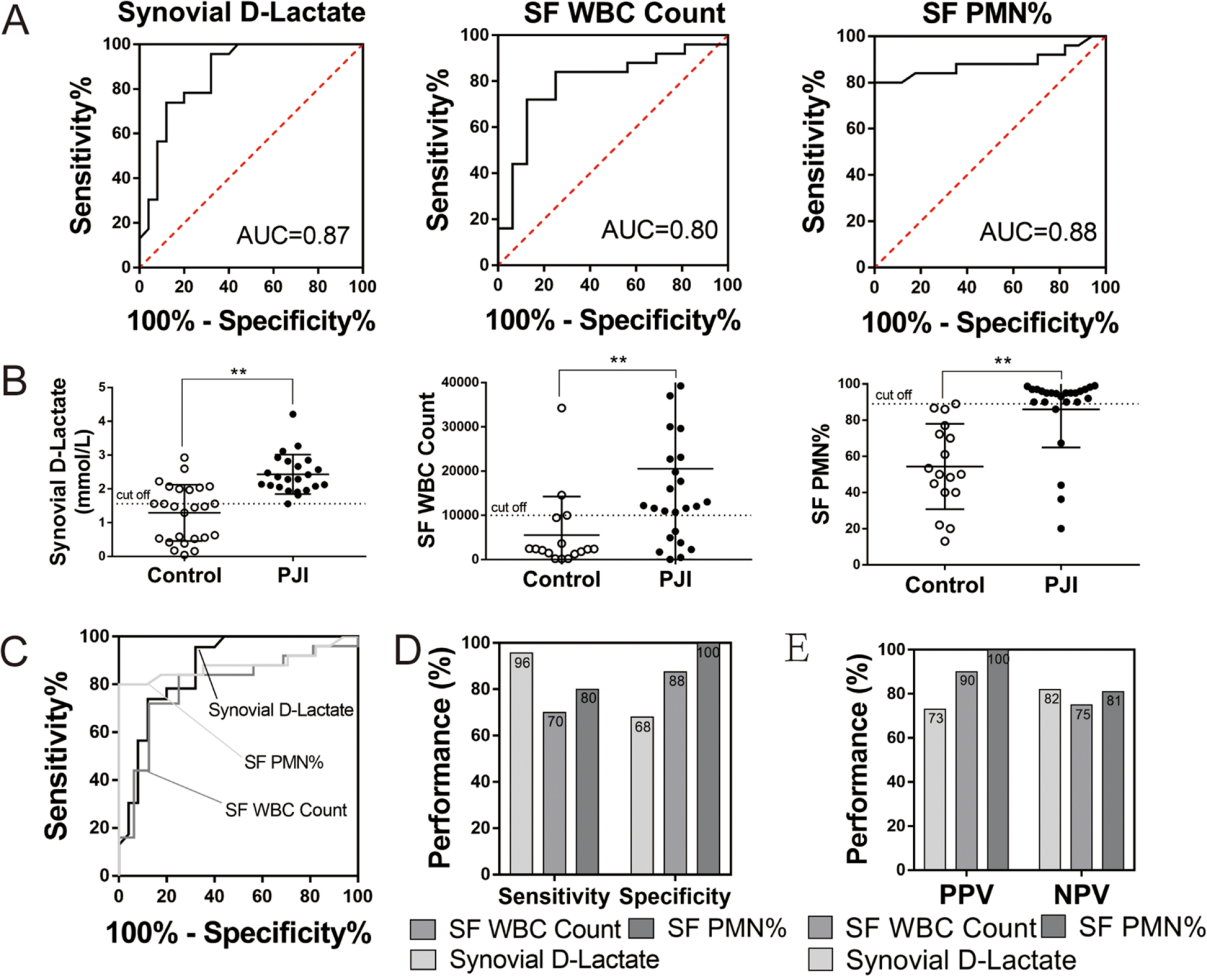
Comparison of synovial D-lactate with leukocyte count and polymorphonuclear neutrophil percentage. **A** and **C** The ROC curves of synovial biomarkers for the diagnosis of PJI. The AUC of synovial D-lactate, SF WBC count and SF PMN%. 0.87, 0.80 and 0.88, respectively. **B** The distributions (median and IQR) of synovial D-lactate, SF WBC count and SF PMN% in PJI and aseptic group. Dotted lines represent the optimal. Threshold based on the current study. **D** Sensitivity and specificity of synovial D-lactate, SF WBC count and SF PMN%. **E** PPV and NPV of synovial D-lactate, SF WBC count and SF PMN%

Synovial markers of synovial D-lactate, SF WBC count and SF PMN% are depicted in Table 6 and Fig 2. The AUC curves for synovial D-lactate, SF WBC count and SF PMN% were 0.87, 0.80, and 0.88, respectively. The optimal predictive cutoff of synovial D-lactate for the diagnosis of PJI was 1.56 mmol/L (sensitivity 95.65%, specificity 68.00%, PPV% 73.33 and 81.82%), whereas the optimal predictive cutoffs for SF WBC count and SF PMN% were 9972.00 and 89.00%, respectively, demonstrating a sensitivity, specificity, NPV and PPV of 70.00%, 87.50%, 90.00% and 75.00% and 80.00%, 100.00%, 100.00% and 81.25%, respectively.

**Table 6:** Performance of biomarkers from synovial fluid for diagnosing PJI

	AUC (95% CI)	Youden index (95% CI)	Optimal Cut-off (95%CI)	Sensitivity (%)	Specificity (%)	PPV (%)	NPV (%)
Synovial D-lactate (mmol/L)	0.87 (0.74 - 0.95)	0.64 (0.38 - 0.76)	1.56 (1.01 - 2.07)	95.65	68.00	73.33	81.82
Synovial fluid white blood cell count (cells/μl)	0.80 (0.64 - 0.91)	0.60 (0.26 - 0.78)	9972.00 (3600.00 – 14536.00.00)	70.00	87.50	90.00	75.00
Polymorphonuclear neutrophil percentage	0.88 (0.75 - 0.96)	0.60 (0.60 - 0.92)	89.00 (86.60 – 89.00)	80.00	100.00	100.00	81.25

*PJI* Periprosthetic joint infection, *AUC* Area under the curve, *CI* Confidence interval, *PPV* Positive predictive value, *NPV* Negative predictive value

### Comparison of serum D-lactate and synovial D-lactate

**Fig. 3:**
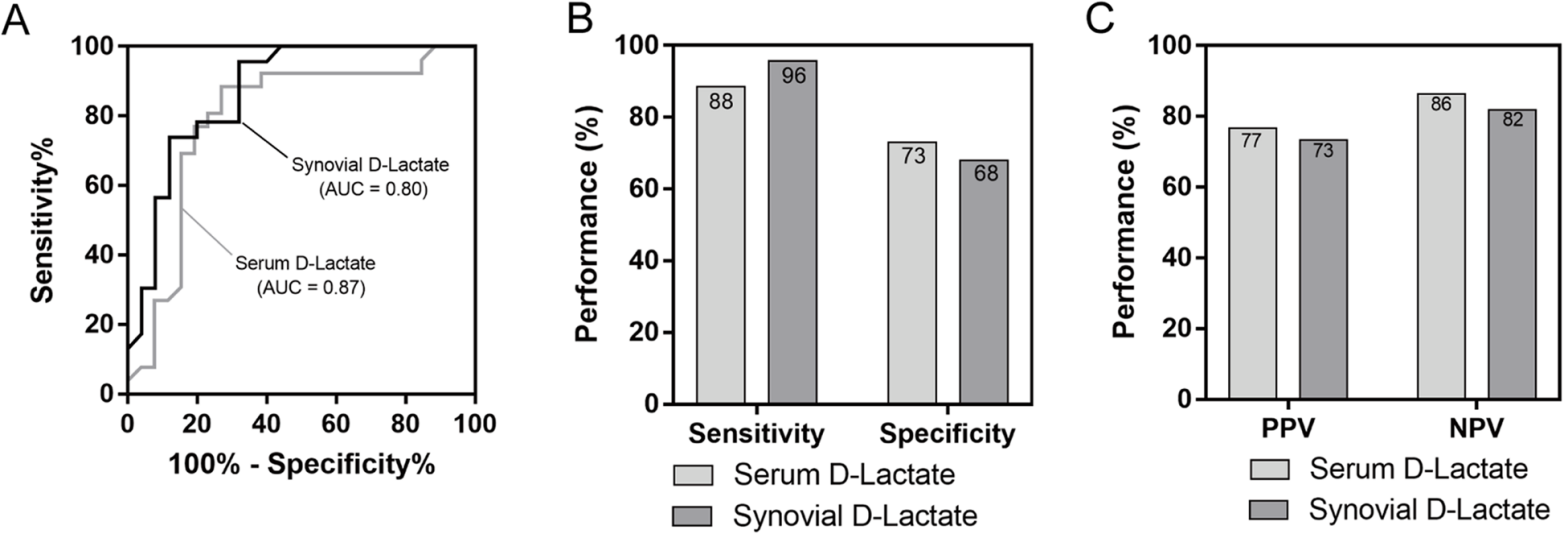
Comparison of serum D-lactate with synovial D-lactate. **A** The ROC curves of serum and synovial D-lactate for PJI. The AUC of serum and synovial D-lactate 0.87 and 0.80, respectively. **B** Sensitivity and specificity of serum and synovial D-lactate. **C** PPV and NPV of serum and synovial D-lactate

In the PJI group, the level of synovial D-lactate was obviously higher than that of serum D-dimer (*p* < 0.05), while the ROC curve showed that synovial D-lactate also had a greater AUC, at 0.88; thus, 1.56 mm/L was chosen as the optimal threshold. When using the optimal threshold to detect synovial D-lactate, the sensitivity of 95.65% was apparently higher than that of serum D-dimer, which was 88.46%, in contrast with its lower specificity, PPV and NPV (68.00%, 73.33% and 81.82%, respectively) than those of serum D-dimer (73.08%, 76.70% and 86.40%, respectively); However both struggle to be highly effective in predicting PJI, especially in terms of high sensitivity (Fig 3A, B and C).

## Discussion

The identification of serological markers to improve the diagnostic efficacy for the first stage of MSIS criteria was carried out [6, 8]. Studies show that in the postoperative period, the ESR and CRP levels are not always reliable to predict treatment failure [6, 22]. However, previous studies have paid the most attention to inflammatory and coagulation biomarkers, rarely focusing on pathogen-related indicators. In our study, we found that serum D-lactate and FIB have potential value for predicting PJI, especially serum D-lactate, with its high sensitivity, whereas the performances of D-dimer, FDP and other indicators (PT, INR, APTT) were poor.

In a previous study, SHARON et al [15, 23] first used D-lactate as a marker to distinguish bacterial infections in rat blood and human body fluids, including ascitic fluids, pleural fluids and cerebrospinal fluids. Then, Gratacos et al [24] demonstrated that D-lactate would be useful to differentiate bacterial synovitis from noninfectious arthritis, choosing a cutoff value of 0.05 mm/L, with a sensitivity and specificity of 85% and 96%. According to our data, serum D-lactate also had better sensitivity and NPV than ESR and FIB, suggesting D-lactate as a good screening test in situations in which the ESR and CRP may be falsely high or low. The 7 false-positive cases could not be explained definitively. Two reasons might account for this result. First, it might be influenced by hemoglobin because insufficient centrifugation and erythrolysis may cause false-positive D-lactate tests due to the similar absorbance wavelengths of 570 nm for D-lactate and 540 nm for hemoglobin [25]. Second, the complications of diabetes (2 cases), hypoproteinemia (1 case) and cervical cancer (1 case) might cause mild glucose metabolic disorders, which lead to pyruvic aldehyde increasing later and ultimately converting to D-lactate in trace amounts [26].

The FIB achieved similarly good performance in diagnosing PJI before revision arthroplasty. The sensitivity and specificity were 80.77% and 73.08%, respectively, when choosing 3.27 g/L, which was in line with previous studies in various research groups [27, 28]. Rui et al [12] reported that an FIB threshold of 4.01 g/L had a sensitivity of 76.3% and a specificity of 86.30%. Nevertheless, the sensitivity and NPV for predicting PJI of ESR and FIB were less prominent than those of serum D-lactate. Meanwhile, as FIB is routinely analyzed before surgery, the time when we would almost be able to confirm the diagnosis of PJI is not practical in first-line screening for PJI in outpatient wards.

FDP, produced when plasmin degrades fibrin and fibrinogen, is related to the activation of fibrinolytic systems. Although Wang et al [29] indicated that FDP analysis would provide diagnostic support for PJI, our result (AUC: 0.69) indicated that the value of FDP for diagnosing PJI is poor. However, the wax and wane with fluctuations of sensitivity and specificity in the predictive cutoff values may be influenced by the sample sizes, genus and species of organisms, patient selection, study biases and clinical laboratory standardization [30].

Regarding D-dimer, we obtained the opposite outcomes (sensitivity 76.00%, specificity 60.00%, PPV% 65.52 and NPV 69.57%) of plasma D-dimer compared with the finding of Shahi, who insisted that serum D-dimer had great value (sensitivity 89.47%, specificity 92.75% PPV% 83.61% and 95.52%) in the assessment of the diagnosis of PJI, with only limited studies further validating this conclusion [31]. Although D-dimer is already one of the novel definitions of periprosthetic joint infection, a number of studies have demonstrated that D-dimer is of limited value for diagnosing PJI [8, 27, 28]. Two factors might contribute to the appearance of substantial discrepancies. First, D-dimer was detected using plasma instead of serum in most medical facilities, while Shahi and colleagues chose serum to determine the level of D-dimer [32]. The difference between plasma and serum may play a role. Second, our patients were mostly Asian, rather than patients in the American population, which is predominantly Caucasian and African American. Different levels of D-dimer in diverse races have been reported, such as between African American and Caucasian patients, which may contribute to the discrepancy from the studies by Shahi et al. [31, 33] and Parvizi et al.[8] Due to the discrepancy, D-dimer as an indicator of PJI should still be regarded with caution, and further studies should be conducted to identify the value of D-dimer for predicting PJI.

Recently, Katsiaryna et al [25] found that the levels of synovial D-lactate were significantly higher in patients in the PJI group than in those in the aseptic group. Choosing 1.26 mmol/l as the optimal threshold value, the sensitivity of 86.40% of synovial D-lactate outperformed both the SF WBC count and SF PMN% (79.5% and 56.8%, respectively), which was mostly consistent with our studies indicating that selecting 1.56 mmol/L as an optimal predictive cutoff resulted in a sensitivity of 95.65%, compared with the sensitivities of 70.00% and 80.00% for SF WBC count and SF PMN%, respectively. Although synovial D-lactate has better sensitivity than serum D-lactate (95.65% and 88.46%, respectively), it was weaker in specificity, PPV and NPV (68.00%, 73.33% and 81.82%) and (73.08%, 76.70% and 86.40%), respectively. However, when synovial D-lactate fails to diagnose or exclude PJI, there are still other biomarkers in synovial fluid to evaluate, such as SF WBC count, SF PMN%, leucocyte esterase, and interleukin 6, in contrast to the few serological options available. Obtaining synovial fluid is an invasive procedure that not only causes pain but also increases the risk of introducing infection into the joint. In addition, it is not always easy and smooth to aspirate enough synovial fluid, especially in the hip [34]. In summary, synovial D-lactate, the assessment of which involves a sophisticated, invasive process and an increased possibility of joint infection, has limited value as an extensive outpatient screening test.

There are several limitations to this study. First, only a limited number of patients were included from our medical center in the cohort analysis. Hence, the conclusions drawn should be further verified in larger samples and multiple centers. Second, the serum samples were all obtained before revision after antimicrobial therapy; thus, the influence of antibiotics on D-lactate cannot be ruled out. Third, the major limitation is that the increased levels of blood glucose, pyruvic aldehyde and ketone bodies in diabetic patients as well as the hemoglobin, L-lactate, fructose 1,6-diphosphate and glycerol 3-phosphate released after erythrocyte lysis might result in partially false-positive patients [26]. Hence, more efficient and accurate detection means and instruments should be chosen for further analysis.

In conclusion, serum D-lactate is a promising serological biomarker for predicting PJI, at least compared to ESR, FIB and synovial D-lactate. D-dimer and FDP may be of limited value in the diagnosis of PJI. The advantages of high sensitivity, pathogenic specificity, rapid availability of the results, little sample demand, concise and minimally invasive processes and low cost make serum D-lactate extremely useful as a real-time screening test for PJI. The combination with confirmatory synovial fluid biomarkers with higher specificity or improvements in the detection method might potentially improve its specificity.

## Data Availability

The datasets analysed during the current study are available from the corresponding author on reasonable request.

## Abbreviation

PJI: Periprosthetic joint infection
ROC: Receiver operating characteristic
AUC: Area under the curve
FIB: Fibrinogen
MSIS: Musculoskeletal Infection Society
CRP: C-reactive protein
ESR: Erythrocyte sedimentation rate
FDP: Fibrinogen degradation product
TKA: Total knee arthroplasty
THA: Total hip arthroplasty
BMI: Body mass index
SF: Synovial fluid
WBC: White blood cell
PMN%: Polymorphonuclear neutrophil percentage
SD: Standard deviations
PPV: Positive predictive value
NPV: Negative predictive value
PT: Prothrombin time
INR: International normalized ratio
APTT: Activated partial thromboplastin time
TT: Thrombin time
